# Chemsearch: collaborative compound libraries with structure-aware browsing

**DOI:** 10.1093/bioadv/vbab008

**Published:** 2021-07-16

**Authors:** Stephen G Gaffney, Sarah Smaga, Alanna Schepartz, Jeffrey P Townsend

**Affiliations:** 1 Department of Biostatistics, Yale University School of Public Health, New Haven, CT 06510 USA; 2 Department of Chemistry, University of California Berkeley, Berkeley, CA 94705, USA; 3 Department of Molecular & Cellular Biology, University of California Berkeley, Berkeley, CA 94705, USA

## Abstract

**Summary:**

Chemsearch is a cross-platform server application for developing and managing a chemical compound library and associated data files, with an interface for browsing and search that allows for easy navigation to a compound of interest, similar compounds or compounds that have desired structural properties. With provisions for access control and centralized document and data storage, Chemsearch supports collaboration by distributed teams.

**Availability and implementation:**

Chemsearch is a free and open-source Flask web application that can be linked to a Google Workspace account. Source code is available at https://github.com/gem-net/chemsearch (GPLv3 license). A Docker image allowing rapid deployment is available at https://hub.docker.com/r/cgemcci/chemsearch.

## 1 Introduction

Research teams that investigate multiple chemical compounds encounter data management challenges as their compound libraries grow (Hohman *et al.*, 2009). One challenge concerns storage: each compound may be associated with many documents and data files, including protocols, experimental outputs, processing scripts and results summaries. These files must be stored somewhere with adequate space, and arranged in a way that makes them easy to access on demand by people or pipelines. Another challenge is access control which requires a mechanism to distinguish team members, who can be provided ready access to files, from outsiders, who might be denied or given restricted access. For those with access, the next challenge is finding specific compounds given that they can have multiple names and identifiers. The ability to ‘search by structure’—finding compounds that match a molecular structure from user input—becomes essential for large libraries. Structure-based search also makes it possible to identify compounds with similar structures or a particular substructure, providing a powerful way to navigate through the structural space of a compounds library.

Chemsearch provides a solution for each of these challenges, having been built to organize and query the ever-expanding chemical library of the Center for Genetically Encoded Materials (C-GEM)—an NSF Center for Chemical Innovation supported by team collaboration software (Gaffney *et al.*, 2019). Requiring only that you arrange your files in a minimally prescribed hierarchy, Chemsearch can automatically extract the compounds and metadata within a library. Linking a Google Workspace account provides secure storage where any team member can contribute new compounds and data files. Chemsearch features a querying interface with the ability to draw or upload compounds, the choice of similarity or substructure searching and a browsing interface to page through compound summaries. These features provide web browser-based navigation functionality similar to public databases, such as PubChem (Kim *et al.*, 2016).

Chemsearch is agnostic to the process of selecting compounds and to the data associated with each compound, making it compatible with any scientific workflow for studying compounds. In a drug-discovery pipeline, it would serve in a data management role (Frearson and Wyatt, 2010; Willems *et al.*, 2020), as an alternative to commercial products such as CDD Vault and Dotmatics. The library could be constructed by library design tools (Saldívar-González *et al.*, 2020) and exported for specialized analysis and visualization in software such as DataWarrior (Sander *et al.*, 2015) or ChemBioServer (Karatzas *et al.*, 2020).

**Fig. 1.**
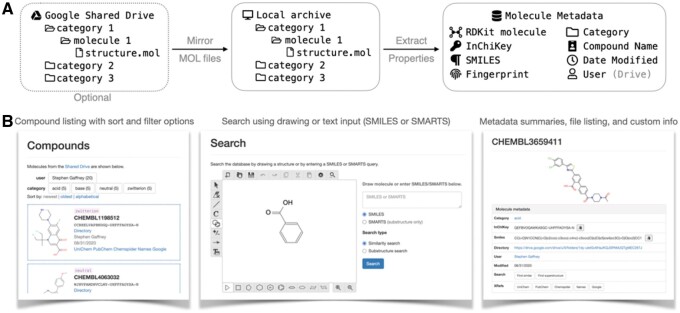
Chemsearch extracts molecular structure from a collection of MOL files, gathers metadata, and provides an interface for browsing and searching the associated compounds. (**A**) Schematic showing initial metadata extraction process, with the expected file hierarchy. (**B**) Screenshots from the Chemsearch web interface

Chemsearch stands out among compound library tools for its team collaboration functionality. There is no need to create an SDF file that stores all structures: structures, names and other compound identifiers are automatically extracted from the Chemsearch file hierarchy, which any user can add to or modify. Team members can store arbitrary experimental data within this hierarchy and collaboratively edit rich-text summary documents for each compound. Chemsearch uses these files to display a file listing and summary HTML. As free and open-source software, Chemsearch also gives teams the ability to customize and extend the tool as they see fit (Fig. 1).

## 2 Features

### 2.1 Cloud storage

Chemsearch can be linked to a Shared Drive, a cloud storage application within Google Workspace, for convenient management of all data associated with the compound library. A Shared Drive lets team members create and organize folders of data files using a web browser, mobile app or the Drive File Stream app for desktop. Shared Drives are included in G Suite for Education, which is free for academic teaching organizations, and in higher-tier Google Workspace versions. Paid accounts charge per user, but a single user will suffice for use with Chemsearch.

Chemsearch uses the Google Drive API, with read-only permissions, to traverse the file hierarchy—indexing the compounds stored within it, and gathering details of associated data files. In this way, the Chemsearch browser lets users explore the library with no risk of accidentally modifying the data. Access privileges for the Drive, meanwhile, are managed within Shared Drive settings, and permit any degree of role-based complexity. Google Groups, part of Google Workspace, provides a simple way to create a list of users (each identified by a Google-associated email address) who should have the same level of access. Such a list can then be given access to the Drive (or a subset of the Drive), assigning a role that specifies permissions—including viewer, commenter, contributor and content manager.

### 2.2 Compound identification

Chemsearch expects the top levels of the compound file hierarchy to follow a fixed pattern to aid identification: (i) the top-level folder contains category folders—a way to offer simple, non-overlapping categorization at the discretion of the team (e.g. ‘aramids’) and (ii) each compound gets its own folder within a category folder and contains an MDL Molfile to store its structure. Additional files associated with compounds can be nested as desired within compound folders.

### 2.3 Metadata extraction

Molfiles are parsed by RDKit (Landrum *et al.*, 2020), an open-source cheminformatics toolkit. RDKit provides alternative representations for each compound (InChiKey, SMILES), calculates structural fingerprints for similarity and substructure matching and generates 2-D images of their structure.

### 2.4 Flexible queries

The search page accepts structural descriptions in the form of a molecular drawing or as a text-based representation, using SMILES (Weininger, 1988) or SMARTS (Daylight Theory Manual) strings. The drawing interface is provided by Kekule.js (Jiang *et al.*, 2016), an open-source client-side script that allows a molecule to be constructed using a palette of atoms and bonds. The script can also load data from a user-supplied Molfile, among other formats. The resulting molecule is converted to SMILES format when the search form is submitted. A valid SMILES string defines a specific molecule, whereas SMARTS—an extension to SMILES—specifies a ‘pattern’ that can be used for matching a range of compounds with desired structural properties. Either SMILES or SMARTS can be used for substructure querying in Chemsearch. SMILES is always used for similarity searches.

### 2.5 Search by structure

For similarity matching, Chemsearch can be configured to use fingerprints and similarity coefficients from a range of options provided by RDKit. The default fingerprint is the Morgan fingerprint (radius 2), selected to approximate the ECFP4 fingerprint that was a top performer in recent benchmarks (O’Boyle and Sayle, 2016). RDKit converts user SMILES input to a fingerprint that is then compared to each of the pre-computed fingerprints in the compound library. A similarity score between 1 (high) and 0 (low), is assigned using a similiarity coefficient, which defaults to the Tanimoto index, a popular similarity coefficient that has performed well in benchmarking studies (Willett, 2006). The results page shows library compounds ordered from most to least similar. Substructure matching is performed by RDKit, which uses an implementation of the VF2 algorithm (Cordella *et al.*, 2004). Chemsearch can be customized to show shortcuts for frequently used substructure patterns: a configuration file with names and SMARTS values is converted to links on the search page.

### 2.6 Browsing

The basic listing view for the homepage and search results displays a grid of summary cards, one for each compound, grouped into pages. Summary cards show a 2D structure representation and key metadata, including name, category, InChIKey, modification date and links to query the InChIKey in external databases. In Shared Drive mode, the uploader and a folder link are also shown. Each compound has a dedicated page containing extended information, including a file listing of the compound folder, and custom data. Custom data can be provided via Google Doc, markdown or other text file in the compound folder, and converted to HTML for display.

### 2.7 Authentication

Authentication mode—which requires a Google Workspace account—provides a mechanism for granting access to specific users, who log in to Chemsearch using Google-associated credentials. Membership is defined by inclusion in a Google Group, making it easy to provision and de-provision access. Authentication mode does not need to be paired with Shared Drive mode, and the Google Group that grants access to the Chemsearch browser is not constrained by which users have direct access to the Drive in Shared Drive mode. In default mode, access is not restricted, so any access control would have to be upstream of the Chemsearch server.

### 2.8 Library management

Specific users can be granted admin privileges, allowing them to access a Chemsearch admin page. The admin page provides the option to rescan the file hierarchy, checking for new molecules and generating metadata, with feedback on progress. The admin page can also be used to export the library to a structure-data file (SDF), and to assign admin status to additional users. The library can also be rescanned from the command line, allowing the process to be triggered manually or by script on a desired schedule.

### 2.9 Open source

Chemsearch is written in Python, using the Flask microframework, and is distributed as free and open-source software on GitHub, under the GPLv3 license. Anyone can make changes to the source code and distribute modified versions with a compatible copyleft license. Components such as the Kekule.js molecule editor could be replaced with alternatives, such as Ketcher (Karulin and Kozhevnikov, 2011), or the proprietary tools MarvinJS (ChemAxon) or ChemdrawJS (PerkinElmer).

## Funding

This work was supported by the Center for Genetically Encoded Materials, an NSF Center for Chemical Innovation [NSF CHE-2002182].

*Conflict of Interest*: none declared.
